# Plasma Protein Comparison between Dairy Cows with Inactive Ovaries and Estrus

**DOI:** 10.1038/s41598-019-49785-8

**Published:** 2019-09-23

**Authors:** Chang Zhao, Shi Shu, Yunlong Bai, Dong Wang, Cheng Xia, Chuang Xu

**Affiliations:** 1 College of Animal Science and Veterinary Medicine, Heilongjiang Bayi Agricultural University, Heilongjiang, 163319 China; 2 Qinghai University, Qinghai, 810000 China

**Keywords:** Proteomics, Animal physiology

## Abstract

To screen differentially expressed proteins in the blood dairy cows with inactive ovaries caused by a negative energy balance and to determine the roles of the identified proteins in the development of inactive ovaries.Holstein cows at 14 to 21 days postpartum in an intensive dairy farm were examined for their energy balance (EB) status by blood β-hydroxybutyrate (BHBA) and assigned to the inactive ovary (IO) group (n = 50) and the normal oestrus control (CON) group (n = 50) at 60 to 90 days postpartum by means of the oestrus manifestation, rectal examination and B-ultrasound examination. Fourteen differentially expressed proteins from 61 proteins in the plasma of dairy cows with IOs were identified by iTRAQ/LC-MS/MS and GO, KEGG, and PATHWAY analysis. Eleven expressed proteins were upregulated, and 3 expressed proteins were downregulated. Among the 10 differentially expressed proteins verified by Western blot or ELISA, the relative expression levels of ALDOB, IGFBP2, ITIH3 and LDHB in mixed samples and single samples were consistent with the proteomic protein results. PKM2, GPX3, ALDOB, RBP4 and AHSG were significantly different between the two groups (*P* < 0.05); APOA4 and SPAM1 were not significantly different (*P* > 0.05) but were still downregulated in the ovarian resting group. This study confirmed that 14 plasma differential proteins in the inactive ovaries of postpartum dairy cows were associated with follicular development, and these findings provide a foundation for further research on the mechanism and prevention of inactive ovaries in dairy cows.

## Introduction

Inactive ovaries in the postpartum period constitutes an abnormal condition in the oestrous cycle of dairy cows, resulting in no follicles on the surfaces of ovaries or no follicular deviation^1^. Inactive ovaries during early lactation in dairy cows are usually classified as Type I anoestrus postpartum based on follicular dynamics, in which the follicles may appear on the surface of the ovary but follicular growth stops^1^. This type of anoestrus increases the number of postpartum days and prolongs the calving interval of dairy cows when the anestrus time is long, from 60 to 90 days postpartum, which may bring about large economic losses to dairy farms. Follicular development in the oestrous cycle of cows is regulated by gonadotropins^2–4^. Gonadotrophin-dependent follicular development is closely related to follicle-stimulating hormone (FSH)^2^, luteinizing hormone (LH), oestradiol (E_2_)^3^ and progesterone (P4)^4^. Nutrition affects the hypothalamic-pituitary-ovarian axis directly through dietary nutrients or metabolic intermediates and then affects follicular development^5^. LH pulse disorder occurs in dairy cows with hypoglycaemia or a negative energy balance^6^. The reason for this association is unclear: if it occurs in the hypothalamus, it is considered to cause LH pulse and ovulation failure by inhibiting GnRH secretion, and if the negative balance affects the ovaries^7^, an increased supply of energy and nutrients can stimulate follicular development^8^. Studies have shown that nutrients act on the ovaries and stimulate follicular development. Glucose, fatty acids and several metabolic hormones can directly stimulate follicles to function^6^.

At present, the proteomic profiles of inactive ovaries postpartum due to negative energy balance (NEB) in dairy cows are unclear. Proteomics technology has been widely used in the dairy industry since the rapid development of new iTRAQ reagents and labelling strategies to expand the identification capacity of differentially expressed proteins by protein bioinformatics for the discovery and elucidation of disease markers^9^. Min *et al*.^10^ applied iTRAQ technology to study heat stress in cows. Dai *et al*.^11^ studied the mechanism of basal feed plus a high feed diet in the regulation of milk synthesis. Zhang *et al*.^12^ used iTRAQ technology to screen differential proteins between the endometrium and plasma of cows with endometritis. In view of the literature and our previous studies, we speculate that certain proteins in the blood of cows may change at 60–90 days postpartum, but to date, these proteins have not been reported. Therefore, in this study, iTRAQ/LC-MS/MS was used to explore differential proteins the blood of dairy cows with inactive ovaries during early lactation to determine the roles of these proteins in the development of inactive ovaries, thus providing a foundation for the establishment of an early warning system regarding inactive ovaries.

## Materials And Methods

### Experimental animals

All methods and animal care were performed in accordance with the relevant guidelines and regulations of the Institute of Animal Science, Chinese Academy of Agricultural Sciences. The study was carried out in an intensive dairy farm of Heilongjiang Province, China, in accordance with the relevant guidelines and regulations of the Institute of Animal Science, Chinese Academy of Agricultural Sciences. The cows were fed a total mixed ration diet during early lactation, which consisted of 8–9 kg of concentrate, 19 kg of silage, 3.5–4.0 kg of hay, and 350 g fat. Their nutritional level on a DM basis included 55.60% DM, 16% crude protein, 7.322 MJ∙kg^−1^ net lactation production, 5.60% fat, 39.10% NDF, 20.30% ADF, 180 g of calcium, and 116 g of phosphorus. The basal diet was formulated to meet the nutrient requirements according to the Feeding Standards of Dairy Cattle in China.

**Figure 1:**
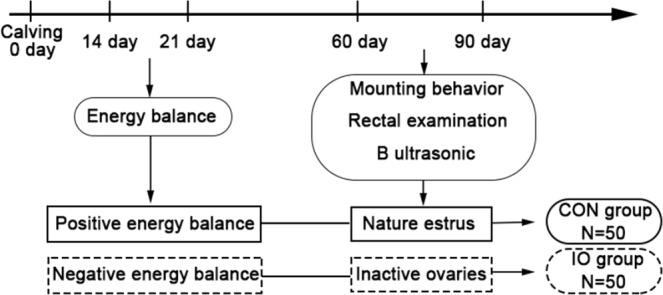
Schematic of the selection of experimental animals.

The experimental animal selection and grouping are shown in Fig. 1 according to the criteria of postpartum inactive ovaries and oestrus in dairy cows^13–15^.

The inactive ovary (IO) group (n = 50): Cows with a high plasma BHBA content (>1.20 mmol/L) at 14–21 days postpartum were in a negative energy balance state, were tracked to 60 days postpartum and had no oestrous signs^13,14^. A rectal examination and B-ultrasound examination were performed at 60–90 days postpartum by a trained professional; if the cow had no follicular development or follicular retardation (less than 4 mm) for 7 days^13,15^, it was assigned to the IO group, and then, all background data, including age, parity, milk yield, disease, and body condition, were collected. If these cows underwent oestrus between 60 and 90 days postpartum and experienced other diseases after calving, they were removed from the IO group.

The normal control oestrus (CON) group (n = 50): Cows with a low plasma BHBA content (>1.20 mmol/L) at 14–21 days postpartum were in a positive energy balance state, tracked to 60 days postpartum and had spontaneously oestrous signs^13,14^. The results of the rectal examination, B-ultrasonography, and follicular diameter measurements of the oestrous cows were the opposite of those in the cows with inactive ovaries^13,15^. The oestrous cows were assigned to the CON group, and then, the background data collected for the IO group were also collected for the CON group. If these cows had other diseases after calving, they were removed from the CON group.

The background data of the two groups of cows, including days in milking, follicular diameter (mm), parity, number of artificial inseminations, body condition score, milk yield (kg/day) 60–90 days after calving, and BHBA (mmol/L) at 14–21 days and 60–90 days after calving, are shown in Table 1.

**Table 1:** Basic information for the experimental animals in the two groups of cows.

Parameters (Mean (SD))	IO (N = 50)	CON (N = 50)	*P*
Days of milking	72.90 (8.322)	73.82 (9.064)	0.735
Follicular diameter (mm)	2.25 (1.37)	10.82 (2.30)	0.000
Parity	3.06 (1.21)	3.19 (1.35)	0.755
Number of artificial inseminations	1.75 (0.91)	1.19 (1.06)	0.607
Body condition score	2.66 (0.25)	2.80 (0.18)	0.053
Milk yield (kg/day)	34.16 (12.53)	33.04 (7.11)	0.721

The inactive ovary group (IO); the healthy normal oestrous control group (CON).

### Sample collection

Blood samples from the two groups of dairy cows were collected via the tail vein before morning feeding. Ten millilitres of blood samples per cow were collected with EDTANa_2_ and centrifuged at 3000 × g/min for 10 minutes to obtain plasma samples. Fifteen plasma samples from each group were used in the proteomics analysis, while the other 35 blood samples were allocated to biochemical analysis and verification of plasma differential proteins by ELISA or Western blot (WB).

### Biochemical analysis

The BHBA, AST, NEFA, and GLU levels in the plasma of the two groups of cows were measured by commercial test kits (CHINA, Yicheng Company) and an automatic biochemical analyser (CHINA, Nanjing Jiancheng Bioengineering Institute).

### iTRAQ labelling proteomics experiments

Five plasma samples from each group were mixed to become a mixed sample, to which lysis buffer was added. The mixed plasma sample was placed under sonication for 60 seconds with a frequency of 0.2 seconds and an amplitude of 22%, extracted at room temperature for 30 minutes, and centrifuged at 20 °C for 20 minutes at 15,000 × g. The supernatant was then carefully collected to determine its protein concentration by the Bradford method^15^. In this experiment, a human plasma/plasma high abundance protein kit (MERCK, USA) was used to remove high abundance proteins in the mixed supernatant samples for iTRAQ-labelling proteomics experiments. Three mixed samples per group (IO group: IO1, IO2, and IO3; CON group: CON1, CON2, and CON3) were used as biological replicates at two time points; the IOs were 116–118, and the CONs were 114–115. Protein solution (200 μg) from a mixed supernatant sample was used to perform proteolysis according to the instructions of the iTRAQ-labelling kit (4352135, AB Sciex).

### LC-MS/MS mass spectrometry analysis

#### Pre-separator

Reversed-phase chromatography separation under high pH conditions: The mixed labelled sample with 100 μL of solution (98% ddH_2_O, 2% acetonitrile, pH = 10) was centrifuged at 14,000 × g for 20 minutes to obtain the supernatant for separation. Separation was carried out using 400 μL of enzymatically decomposed BSA (column temperature, 45 °C; detection wavelength, 214 nm). A total of 100 μL of the prepared sample was loaded at a flow rate of 0.7 mL/min^16^.

Nanoscale reversed-phase chromatography-Q Exactive for protein analysis: The fraction obtained by reversed-phase separation under high pH conditions was reconstituted with 20 μL of 2% methanol and 0.1% formic acid and centrifuged at 12,000 × g for 10 minutes. The supernatant was then drawn to load 10 μL of the supernatant sample. The loading pump flow rate was 350 nL/min, for 15 minutes, and the separation flow rate was 350 nL/min^15^.

### Data analysis

SPSS software (IBM, V 20.0) was used to compare the basic information of the two groups of experimental cows and their blood sample results by means of the independent sample t-test.

Mass spectrometry of iTRAQ was performed by Thermo Q-Exactive mass spectrometry, and the resulting mass spectrometry raw files were processed using the commercial software Proteome Discoverer 1.4 (Thermo) and the UniProt database (http://www.uniprot.org). The differential proteins were screened using mass spectrometry software (Thermo) based on a 5% FDR^17^.

### Functional analysis of differentially expressed proteins using bioinformatics

This study used the DAVID (https://david.ncifcrf.gov/) online data software for GO analysis, the STRING (https://string-db.org/) online software for the protein network interaction analysis and, mainly, the KEGG Pathway database application with the DAVID online data software for signalling pathway analysis^18^.

### Differential protein verification

Differential proteins screened by the proteomics and bioinformatics experiments were verified by an Western Blot (WB) analysis, in which a differential protein was resolved in one lane of a gel. As in the proteomics experiments, six mixed samples was used in WB analysis. The WB experiment was performed according to the conventional method^19^. Based on the bioinformatics analysis of differential protein function, four representative differential proteins were selected for WB verification, in which 30 μg per sample was loaded in a lane for WB experiments. The verification of a differential protein was conducted be means of gel electrophoresis; one gel contained 9 lanes, in addition to the lane for the protein marker. For the experiment, 6 mixed samples was used to WB analysis. The differential proteins included fructose-bisphosphate aldolase (ALDOB), insulin-like growth factor binding protein (IGFBP-2), inter-alpha-trypsin inhibitor heavy chain H3 (ITIH3), and L-lactate dehydrogenase (LDHB), which were immunoblotted using respective monoclonal antibodies (18065-ap-1 ALDOB antibody from US Proteintech, US; ab4244 IGFBP-2 antibody from UK Abcam, 21247-ap-1 ITIH3 antibody from US Proteintech, and SAB2101329 LDHB antibody from Japan SIGMA). The density of the grey bands of differential proteins in the WB were calculated using ImageJ2x (Rawak Software, Inc. Germany) and subjected to statistical analysis by the independent sample t-test in SPSS software (IBM, V20.0).

Commercial ELISA kits were used to measure the concentrations of the five differentially expressed proteins in 15 untreated plasma samples per group. These differential proteins were α-2-HS glycoprotein (JEB-15398 ELISA kit), apolipoprotein A4 (JEB-15400 ELISA kit), fructose diphosphate aldolase (JEB-15230 ELISA kit), glutathione peroxidase, hyaluronidase (JEB-15220 ELISA kit), pyruvate kinase and retinol binding protein (JEB-15397 ELISA kit). These ELISA kits were from Nanjing Jinyibai Company in China. Differences between the two groups were compared using SPSS software (IBM, V20.0) and the independent sample t-test.

### Innovation statement

Inactive ovaries in postpartum dairy cows constitute an economically important reproductive condition that can reduce the efficiency of cow production and reproduction, and this condition has garnered a large amount of attention in the dairy industry of many developed countries. If any step after calving is abnormal between the follicular growth, selection and ovulation stages and fertilization and pregnancy stages, infertility may ensue. The literature and our previous studies have found that a negative energy balance in postpartum cows can increase the rate of anestrus at 50–60 days postpartum, which may be related to metabolic disorders, such as abnormal metabolism of lipids, amino acids and steroids. The authors of this paper have put forth the hypothesis that the expression of some proteins in the blood of cows with inactive ovaries are different from that of normal oestrus cows at 60–90 days after calving. Thus, in this study, proteomics technology was used to screen differential proteins in the blood of cows with inactive ovaries. Our study found that in the blood of cows with inactive ovaries at 60–90 days postpartum, there were changes in 61 proteins, 14 of which were related to inactive ovaries.

## Results

### Background information and biochemical parameters

According to the background information of the CON group and IO group in Table 1, there was no significant difference in days of milking, age, number of artificial inseminations, body condition score, or milk yield between the two groups of cows. However, the plasma biochemical parameter levels of the inactive ovary group (IO) and the healthy control group are shown in Table 2. There was a significant difference in the plasma BHBA concentration between the two groups of cows at 14–21 days postpartum, suggesting that the cows in the IO group experienced a negative energy balance at 14–21 days postpartum, although there was no difference in plasma BHBA levels of the two groups of cows at 60–90 days after calving. The energy balance in the two groups of dairy cows is briefly summarized in Fig. 1.

**Table 2:** Plasma levels of energy and liver function parameters in the two groups of cows.

Indicator	IO (N = 35)	CON (N = 35)	*P*
AST (U/L)	92.06 ± 17.95	98.94 ± 23.26	0.189
Glu (mmol/L)	3.75 ± 1.40	3.65 ± 0.55	0.686
NEFA (mmol/L)	0.21 ± 0.17	0.26 ± 0.15	0.150
BHBA (mmol/L) (14–21days postpartum)	1.56 (0.14)	0.57 (0.22)	0.000**
BHBA (mmol/L) (60–90 days postpartum)	0.61 (0.14)	0.48 (0.16)	0.073

β-hydroxybutyrate (BHBA), Aspartate aminotransferase (AST), glucose (GLu), Nonesterified fatty acid (NEFA).

### Screening and identification of differential proteins

In this study, a calculation of the average difference of multiples was used to screen differential proteins. In Table 3, 61 differentially expressed proteins were obtained according to a mean fold change >1.2, mean fold change <0.8, and p < 0.05. The proteins with a mean fold change >1 were upregulated in the IO group, while the proteins with a mean fold change <1 were downregulated.

**Table 3:** Differentially expressed proteins between the two groups of cows.

NO.	ID	ABBREVIATION	MFC	U/D	*P* value
1	Q2TBU0	HP	0.36	D	0.000
2	A0JNP2	SCGB1D	0.48	D	0.000
3	P23805	CGN1	0.54	D	0.000
4	P02676	FGB	0.55	D	0.000
5	A5PJE3	FGA	0.56	D	0.000
6	Q3SZZ9	FGG	0.58	D	0.000
7	E1BJF9	SAA	0.62	D	0.000
8	Q3SYR8	IGJ	0.63	D	0.000
9	O97941	C3	0.65	D	0.000
10	A6QQ07	BTD	0.65	D	0.003
11	A6QNW3	PIGR	0.69	D	0.000
12	A5D984	PKM2	0.69	D	0.000
13	P01966	HBA	0.70	D	0.000
14	G3X8D7	GPX3	0.70	D	0.000
15	Q9TS74	PEI	0.71	D	0.000
16	Q17QH1	APOF	0.72	D	0.000
17	A5D9D2	C4BPA	0.73	D	0.000
18	Q9BGI3	PRDX2	0.74	D	0.000
19	K7QM77	ATP6	0.75	D	0.000
20	F1MR06	ATP1A3	0.78	D	0.020
21	P01030	C4	0.78	D	0.019
22	Q1RMN8	IGL	0.79	D	0.000
23	Q6Q0I7	F11	0.79	D	0.011
24	Q3SZZ7	FGL1	0.79	D	0.000
25	M5FJT7	GNPTG	0.80	D	0.016
26	A6QNW7	CD5L	0.80	D	0.000
27	Q32L76	SAA4	0.80	D	0.000
28	F1MXS8	COL3A1	0.81	D	0.005
29	Q3ZCH5	AZGP1	1.20	U	0.000
30	Q3Y5Z3	ADIPOQ	1.20	U	0.000
31	F1MTT3	F12	1.20	U	0.000
32	P56652	ITIH3	1.21	U	0.000
33	F1N3Q7	APOA4	1.21	U	0.000
34	A9UIB1	PON1	1.23	U	0.000
35	P80195	GLYCAM1	1.23	U	0.001
36	P18902	RBP4	1.24	U	0.000
37	A5PK73	ALDOB	1.24	U	0.000
38	P34955	SERPINA1	1.24	U	0.000
39	P15467	RNASE4	1.26	U	0.000
40	A8DBT6	CD14	1.27	U	0.000
41	Q9XSG3	IDH1	1.28	U	0.008
42	Q862Q0	SPM	1.28	U	0.002
43	F1N2P8	IGFBP2	1.29	U	0.000
44	B0JYN3	LDHB	1.29	U	0.000
45	Q3SZH5	AGT	1.31	U	0.000
46	P12763	AHSG	1.32	U	0.000
47	P19034	APOC2	1.32	U	0.000
48	K4JDT2	A2M	1.36	U	0.000
49	F1MNV5	KNG1	1.37	U	0.000
50	P33433	HRG	1.38	U	0.013
51	Q28065	C4bBPA	1.42	U	0.000
52	P25326	CTSS	1.43	U	0.000
53	K7QF89	BoLA	1.43	U	0.000
54	Q58D62	FETUB	1.49	U	0.000
55	Q58DL9	PLTP	1.51	U	0.008
56	O02659	MBL	1.60	U	0.001
57	Q0VCM4	PYGL	1.64	U	0.003
58	D4QBB3	HBB	1.74	U	0.000
59	F1MTV1	SPAM1	1.87	U	0.000
60	Q3MHN5	GC	2.86	U	0.000
61	Q8JTX9	LW012	6.11	U	0.000

MFC: Mean fold change; U: upregulated expressed proteins: D: downregulated expressed proteins.

### Identification of key differentially expressed proteins associated with inactive ovaries

**Figure 2:**
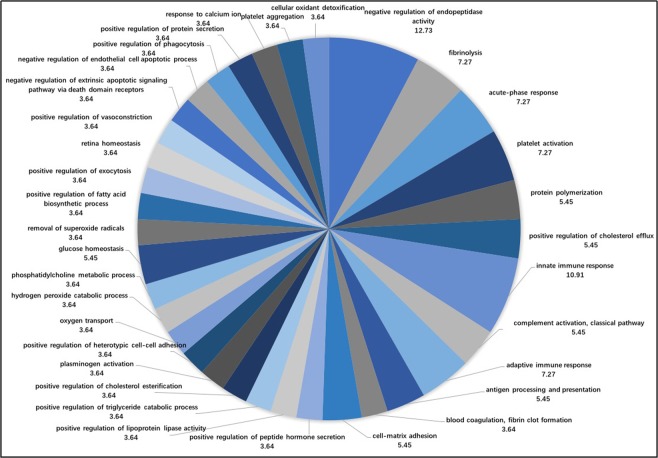
The proportions of accessions from the biological process analysis.

Figure 2 shows that the proportion of accessions from the Biology Process analysis included 34 annotations. In the GO analysis, the biological processes with higher proportions were negative regulation of endopeptidase activity (12.73%), innate immune response (10.91%), fibrinolysis (7.27%), acute phase response (7.27%), and platelet activation (7.27%).

**Figure 3:**
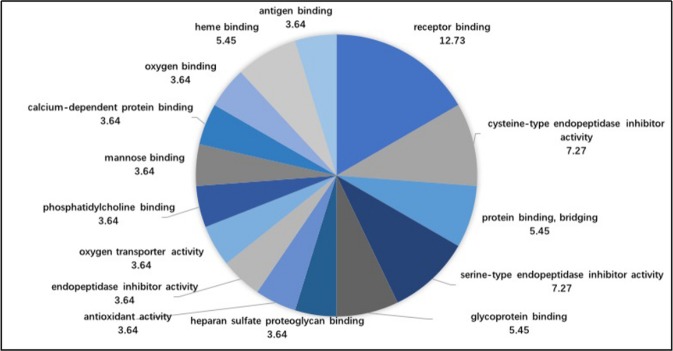
The proportions of accessions from the molecular function analysis.

Figure 3 shows that the molecular function analysis included 15 annotations. The five biological processes were receptor binding (12.73%), cysteine endopeptidase inhibitor activity (7.27%), serine endopeptidase inhibitor activity (7.27%), protein binding (5.45%) and glycoprotein binding (5.45%).

**Figure 4:**
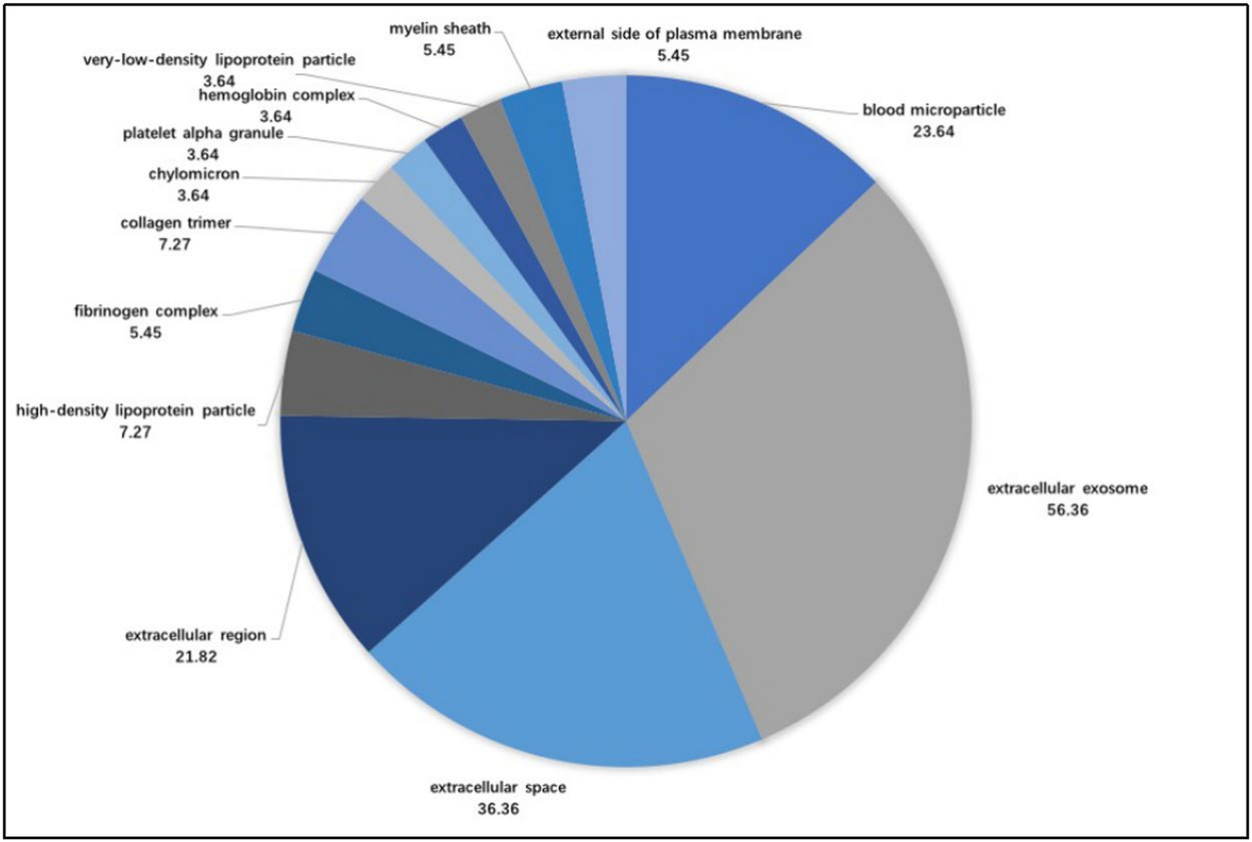
The proportions of accessions from the cellular component analysis.

Figure 4 shows that the cell composition included 13 annotations. The biological processes with higher proportions were extracellular exosome (56.36%), extracellular space (36.36%), blood, microparticles (23.64%), extracellular regions (21.82%) and high-density lipoprotein particles (7.27%).

**Figure 5:**
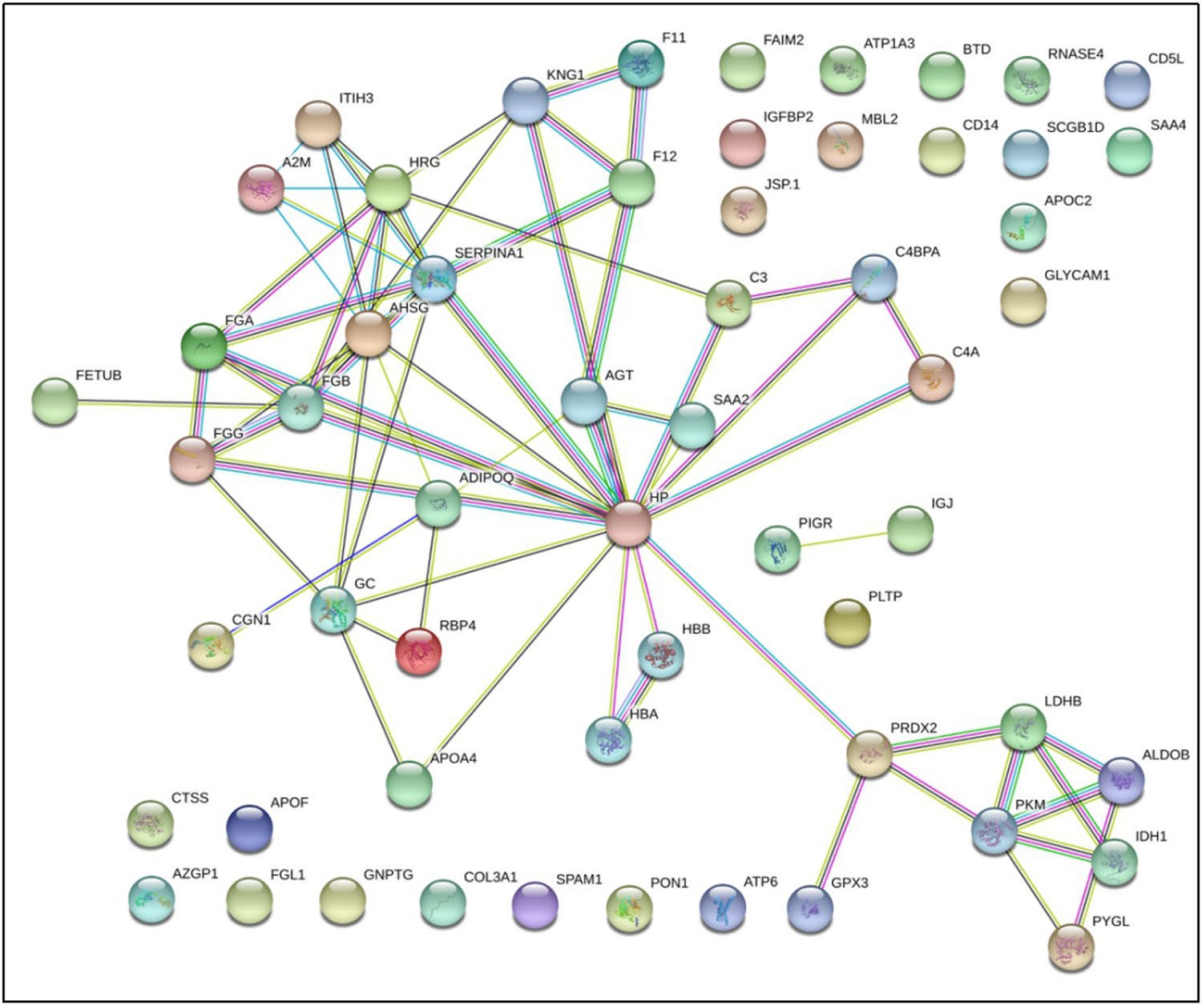
The network interaction diagram of the differentially expressed proteins. The interaction map contains only differentially expressed proteins, resulting in 57 nodes, 68 protein interactions, and a protein interaction enrichment of p < 10–16. The interrelated centre is the interaction between HP and PRDX2.

The protein network interactions of 61 differential proteins are shown in Fig. 5. Three proteins were not matched in the database (PEI, SPM and LW012), 57 nodes were obtained, and 68 proteins were annotated with each other. The proteins with *p* < 10^*−16*^ were enriched.

Based on the bioinformatics analysis of 61 differential proteins (including the GO, protein interaction and Pathway analyses), as well as the search of literature and data related to differential proteins, 14 differential proteins related to inactive ovaries were identified, including three downregulated proteins, namely, GPX3, SCGB1D and PKM2, and 11 upregulated proteins, namely, ADIPOQ, AHSG, APOA4, FETUB, ALDOB, SPAM1, LDHB, RBP4, IGFBP2, ITIH3 and GLYCAM1.

**Figure 6:**
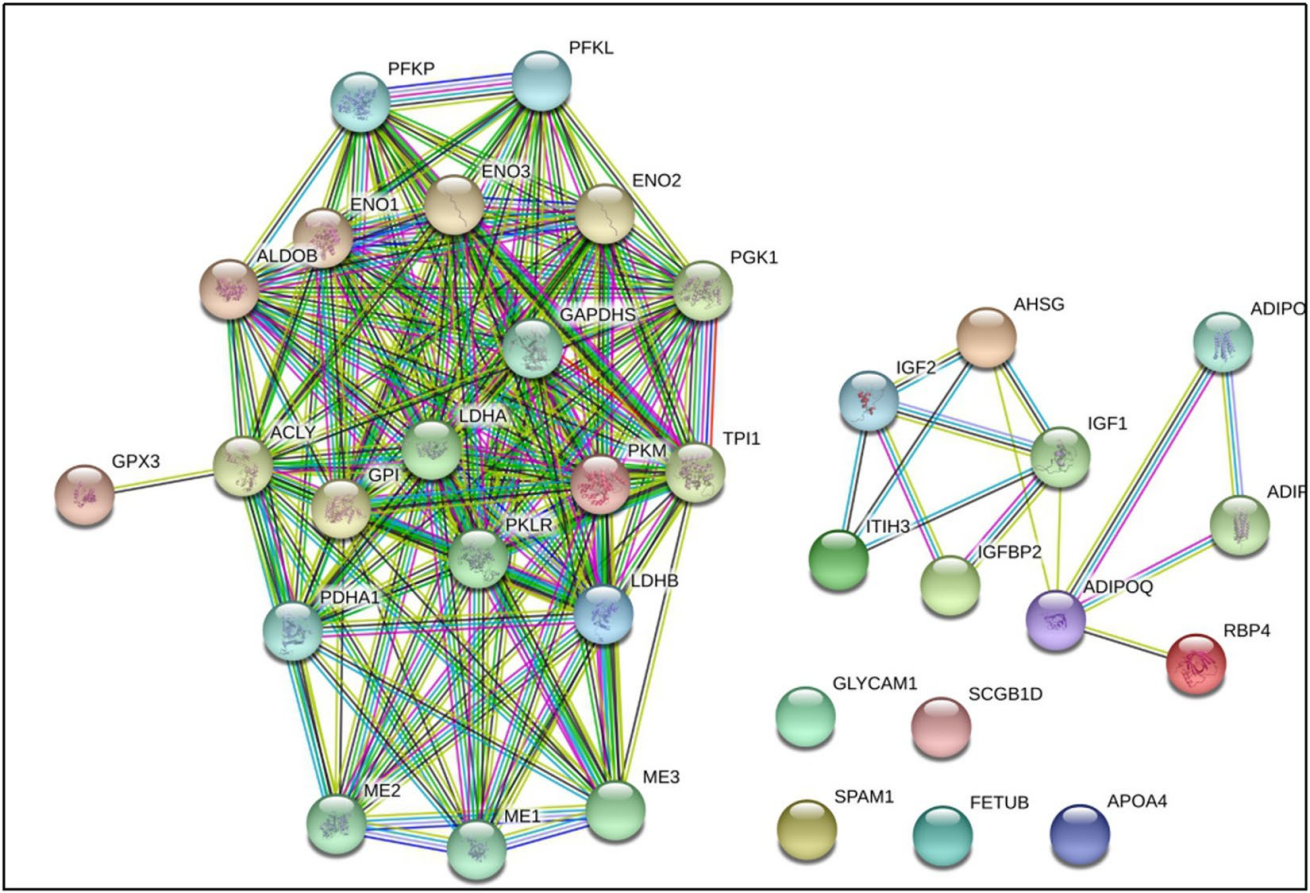
The network interaction diagram of 14 key proteins. Fourteen differentially expressed proteins were screened and analysed, including three downregulated proteins, namely, GPX3, SCGB1D, and PKM2, and 11 upregulated proteins, namely, ADIPOQ, AHSG, APOA4, FETUB, ALDOB, SPAM1, LDHB, RBP4, IGFBP2, ITIH3, and GLYCAM1. The differential proteins aggregated on the left belong to the interaction of proteins in the sugar metabolism pathway. The IGFBP2 protein and the IGF family of proteins are found on the right side, suggesting that the related differential proteins are involved in the secretion, metabolism, and function of reproductive hormones.

Figure 6 shows that the interaction centre is HP and PRDX2. The pathway analysis of differentially expressed proteins is shown in Table 4 and includes 11 metabolic pathways. After bioinformation research was performed, it was found that glycolysis/gluconeogenesis, amino acid biosynthesis, the glucagon signalling pathway, and vitamin digestion and absorption may be associated with inactive ovaries.

**Table 4:** Signalling pathway analysis of the differentially expressed proteins.

KEGG ID	Description	Count	*p* value	Proteins
bta04610	Complement and coagulation cascades	10	0.000	C4bBPA/KNG1/C4/FGB/FGG/F12/MBL/C4BPA/FGA/SERPINA1/A2M
bta04611	Platelet activation	4	0.016	FGB/FGG/FGA/COL3A1
bta05150	Staphylococcus aureus infection	3	0.025	FGB/FGG/MBL
bta00010	Glycolysis/Gluconeogenesis	3	0.029	ALDOB/PKM2/LDHB
bta04145	Phagosome	4	0.030	CGN1/MBL/CTSS/CD14
bta01230	Biosynthesis of amino acids	3	0.039	IDH1/ALDOB/PKM2
bta05133	Pertussis	3	0.041	C4bBPA/C4/C4BPA/CD14
bta01130	Biosynthesis of antibiotics	4	0.055	IDH1/ALDOB/PKM2/LDHB
bta04922	Glucagon signaling pathway	3	0.060	PYGL/PKM2/LDHB
bta01200	Carbon metabolism	3	0.077	IDH1/ALDOB/PKM2
bta04977	Vitamin digestion and absorption	2	0.089	APOA4/BTD

### Proteomics validation

**Figure 7:**
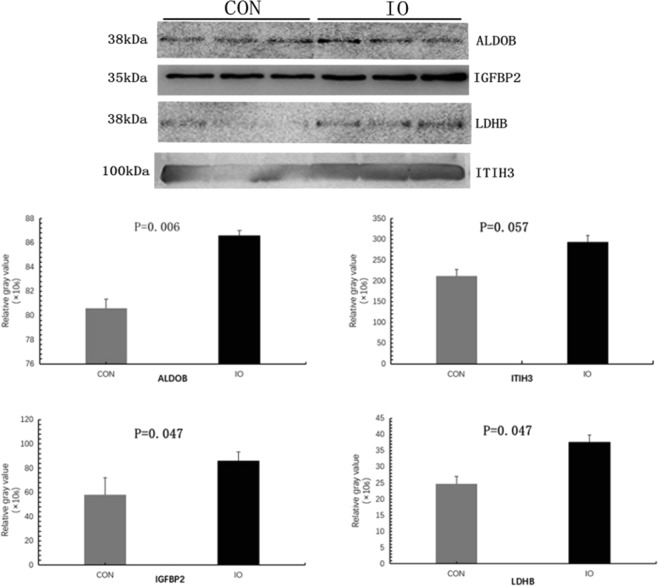
WB and Grey value analysis for ALDOB, IGFBP2, LDHB, and ITIH3. CON is a mixed sample of the healthy normal oestrous control group; IO is a mixed sample of the inactive ovary group.

Four proteins (ALDOB, IGFBP2, ITIH3, and LDHB) from the proteomics analysis were validated by Western blot and protein band gel densitometric analysis, and the grey value analysis is shown in Fig. 7. The relative expression levels of the four proteins in the mixed sample was consistent with the results of the proteomics test.

**Figure 8:**
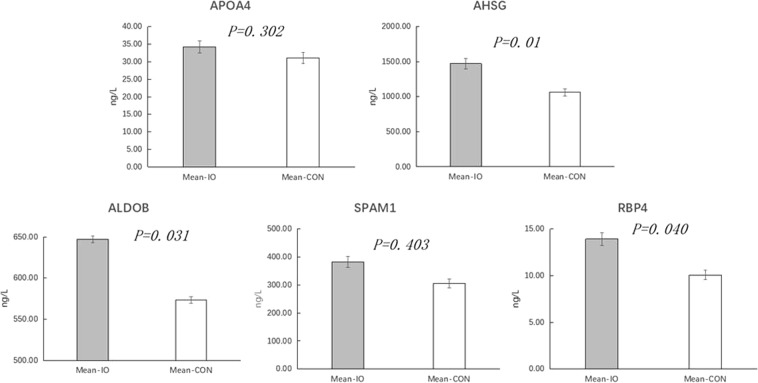
Concentrations of the upregulated AHSG, APOA4, ALDOB, SPAM1 and RBP4 proteins. AHSG is alpha 2-HS glycoprotein; APOA4 is glutathione peroxidase 3; ALDOB is fructose diphosphate aldolase; SPAM1 is hyaluronidase; RBP4 is retinol binding protein 4; Mean-IO is the mean value of the sample in the IO group; and mean-CON is the average of the CON samples. The ordinate is protein content.

**Figure 9:**
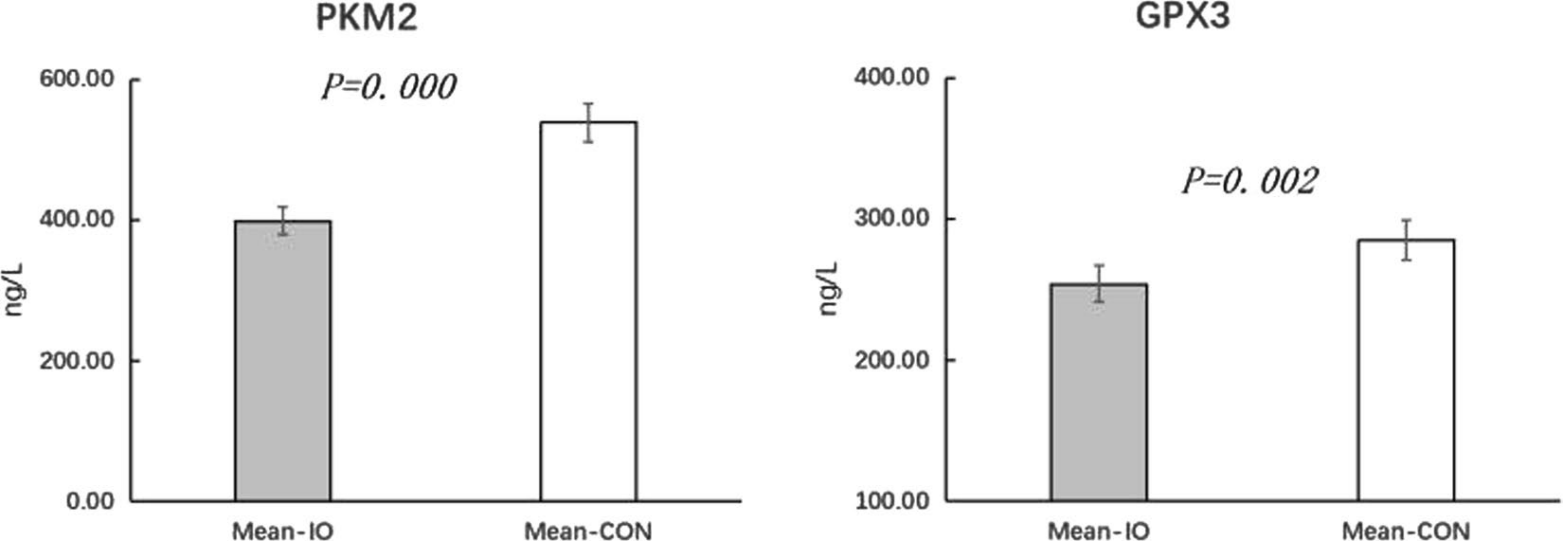
Concentrations of the downregulated PKM2 and GPX3 proteins. PKM2 is pyruvate kinase; GPX3 is glutathione peroxidase 3; Mean-IO is the mean value of the samples from the IO group; Mean-CON is the mean value of the samples from the CON group; and the y-axis is the concentration.

In addition, seven differentially expressed proteins were validated using the ELISA method, as shown in Figs 8 and 9. In the inactive ovary group, PKM2 and GPX3 were significantly downregulated (*P* < *0.01*), AHSG was significantly upregulated (*P* < *0.01*), and ALDOB and RBP4 were significantly upregulated (P < 0.05). However, APOA4 and SPAM1 were not significantly downregulated in the inactive ovary group (P > 0.05).

## Discussion

In this study, iTRAQ/LC-MS/MS was used to identify 14 major differentially expressed proteins in the blood of the inactive ovaries of dairy cows. Ten differentially expressed proteins were verified by WB or ELISA experiments, and the results were consistent with the proteomics results. In the inactive ovary group, the concentrations of ALDOB in the mixed samples and single samples were significantly increased (*P* < *0.01*), the levels of LDHB and IGFBP2 were significantly increased (*P* < *0.05*), and the concentration of ITIH3 was not significantly increased (*P* > *0.05*). Bioinformatics analysis revealed that the four differentially expressed proteins participate in glucose metabolism, especially the metabolic pathway of glycolysis, as blood glucose can play a role in follicular development. Next, the four differential proteins were examined to determine the relationship between the glycolytic process in follicles a ovarian rest.

ADIPOQ is a protein that is mainly produced by white adipose tissue and may regulate ovarian function and early pregnancy^20^. In dairy cows, ADIPOQ and its receptors are present in different cell types, including oocytes, follicular membranes, granulosa cells, cumulus cells, and luteal cells^21^. Moreover, the physiological state of the ovaries is related to the expression pattern of ADIPOQ and its receptor on the surface of follicular cells and luteal cells^22^. In fact, in the follicular phase and the luteal phase, the expression levels of adiponectin, ADIPOQ receptor 1 and ADIPOQ receptor 2 are higher in granulosa cells and cumulus cells in the dominant follicle than in the atretic follicle^23^. ADIPOQ may also reduce the steroid production induced by insulin through the ERK1/2 MAPK pathway, but ADIPOQ only inhibits steroid production in bovine follicular cells^24^. In this study, the plasma level of ADIPOQ was upregulated in dairy cows with inactive ovaries. This suggests that an indirect effect on follicular development may be induced by insulin and IGF-1^25^, which needs further confirmation.

Glutathione peroxidase belongs to a family of phylogenetically related enzymes, and the catalytic centre of mammalian GPX1-4 is selenocysteine. Glutathione is a substrate for GPX3, a major antioxidant enzyme that protects cells from lipid hydrogen peroxide and H_2_O_2_^26^ and catalyses the reduction of free hydroperoxides and other hydroperoxides^27^. Excessive oxidative stress induces apoptosis in follicular cells and induces atresia^28^. GPX3 prevents apoptosis from oxidative stress and promotes follicular growth. In this experiment, the plasma level of GPX3 was downregulated in dairy cows with inactive ovaries, suggesting that oxidative stress in ovarian quiescence may cause the failure of follicular development to form a cumulus structure that promotes oocyte production.

The insulin-like growth factor (IGF) system consists of several members, including IGF-1 and IGF-2, two receptors, and at least six binding proteins (IGFBP2, IGFBP2, IGFBP3, IGFBP4, IGFBP5, and IGFBP6). IGFBPs are present in biological fluids and function by inhibiting or enhancing the actions of two IGFs (IGF1 and IGF2) in target cells^29^. The regulation of the bioavailability of IGFBP by IGFBPs is essential for the *in vitro* culture of bovine oocytes and follicles^30^. The expression of IGF1 receptor in bovine follicular granulosa cells and ovarian follicular cells increases in the final stages of follicular development and decreases at the onset of occlusion^31^. Although IGF1 and insulin play a key role in late follicular development, IGFBP2 expression is upregulated in cows with inactive ovaries, and the follicles under these conditions stop developing at an early stage.

Retinol binding protein 4 (RBP4) is also known as vitamin A binding protein 4^32^. The transport and metabolism of retinoids is mediated and regulated by specific binding proteins, which have the potential to locally regulate follicular development, including oocyte maturation^33^. Retinoic acid is an activated form of retinol and is also an oxidized form. Retinoic acid can reduce the levels of FSH receptors and LH receptors induced by FSH in granulosa cells. The acquisition of LH receptors and FSH receptors is critical for follicular development, oocyte maturation, ovulation and luteinization. Therefore, an increase in vitamin A and its derivatives can affect the development of follicles. In this experiment, the plasma level of RBP4 increased in the cows with inactive ovaries, but the mechanism RBP4 actions in the inactive ovaries of dairy cows needs further exploration in the future.

In summary, among the four major differential proteins, ADIPOQ, IGFBP2 and RBP4 may affect follicular development by influencing the biological processes of reproductive hormones, while GPX3 affects follicular development through oxidative stress. However, it is still unclear how these differential proteins interact in follicular development and inactive ovaries during the early lactation of dairy cows.

## Conclusions

This study is the first to identify 61 types of differentially expressed proteins in the serum of cows with inactive ovaries based on iTRAQ and LC-MS/MS. Among these proteins, 14 were closely related to inactive ovaries; GPX3, SCGB1D and PKM2 were downregulated, and ADIPOQ, AHSG, APOA4, FETUB, ALDOB, SPAM1, LDHB, RBP4, IGFBP2, ITIH3 and GLYCAM1 were upregulated. This implies that the 14 differentially expressed proteins are closely associated with the development of inactive ovaries during early lactation in dairy cows. However, there will be many future research studies on how these differential proteins play a role in the development of inactive ovaries.
